# Intraspecific Hybridization Improves Performance of the Biological Control Agent *Hypena opulenta*

**DOI:** 10.1111/eva.70306

**Published:** 2026-07-31

**Authors:** Brianna Foster, Ruth. A. Hufbauer, Marianna Szűcs

**Affiliations:** ^1^ Department of Entomology Michigan State University East Lansing Michigan USA; ^2^ Invasive Plant Research Laboratory USDA‐ARS Fort Lauderdale Florida USA; ^3^ Department of Agricultural Biology and Graduate Degree Program in Ecology Colorado State University Fort Collins Colorado USA

**Keywords:** biological control, genetic diversity, heterozygosity, *Hypena opulenta*, intraspecific hybridization, swallowwort

## Abstract

Introduced species often undergo genetic drift and natural selection, which can reduce genetic diversity and drive differentiation among populations. Intraspecific hybridization, outcrossing among individuals from genetically distinct groups, may counteract these effects by increasing heterozygosity, potentially improving performance. However, in classical biological control agents, genetic diversity is typically low due to small founding sizes, long‐term quarantine rearing, and single‐source collections from the native range. As a result, opportunities for beneficial hybridization may be limited. We evaluated whether intraspecific hybridization could enhance genetic diversity and performance of *Hypena opulenta* (Lepidoptera: Erebidae), a biological control agent of invasive swallow‐wort vines (*Vincetoxicum* spp.) in North America. We used two populations: one reared under laboratory conditions for 28 generations, and another established in the field in Canada, separated from the lab population for 14 generations. We created a third population by crossing individuals from the two source populations. We conducted Whole Genome Sequencing (WGS) on 15–20 individuals per population to assess genetic divergence and heterozygosity and compared performance of the three populations under both laboratory and field conditions. Despite relatively recent separation, clear genetic structure had developed between the lab and field populations. Both exhibited low genome‐wide heterozygosity, while the outbred population showed significantly higher heterozygosity. Hybridization also led to improved performance: outbred females produced more pupae in the laboratory, caused greater defoliation of host plants, and yielded the most second‐generation adults in the field. These results demonstrate that even short‐term isolation can lead to meaningful divergence and that intentional intraspecific hybridization can boost both genetic diversity and performance. Our findings suggest that outcrossing among recently separated populations could be a practical approach to improve performance of biological control agents prior to field release, with broader implications for managing genetic diversity in applied ecology and conservation contexts.

## 1 Introduction

Evolutionary processes play a critical role in population establishment and persistence in changing environments, particularly for organisms with small populations (Sakai et al. 2001; Szűcs et al. 2014). Small populations lose diversity by drift, which hinders responses to selection, and both drift and inbreeding increase genome‐wide homozygosity. When individuals are homozygous for recessive deleterious alleles, fitness is reduced, and those individuals have high genetic load relative to heterozygotes, though such alleles may also be purged when exposed in the homozygous state. Intraspecific hybridization, the mating of individuals from genetically distinct populations of the same species (i.e., outcrossing or outbreeding), can counter these deleterious processes and improve individual and population performance (Colautti et al. 2017; Frankham et al. 2011; Whiteley et al. 2015). Intraspecific hybridization can increase heterozygosity, thereby masking the effects of deleterious recessive alleles and enhancing genetic diversity for natural selection to act upon, accelerating adaptation to novel environments (Barker et al. 2019; Sinclair et al. 2019; Stewart et al. 2017; Szűcs et al. 2017). These benefits of intraspecific hybridization are increasingly supported by research examining the demographic and genetic processes that shape the success of small populations and often are studied under the context of genetic rescue (Bell et al. 2019; Frankham et al. 2017; Ralls et al. 2018) but also in the context of invasion biology (Ellstrand and Schierenbeck 2000) and biological control (reviewed in Szűcs, Vercken, et al. 2019). In this manuscript, we use intraspecific hybridization to refer to the crossing of genetically distinct populations within a species as an evolutionary process. We use outcrossing and outbreeding to refer to the act of crossing of unrelated individuals from different populations, and outbred population to refer to the resulting offspring. However, there are three situations in which outcrossing may not be beneficial. First, if populations are too divergent, the breakdown of co‐adapted gene complexes or other genetic incompatibilities may occur, leading to outbreeding depression (Wei and Zhang 2018). Second, adaptation of parental populations to different environments can lead to poor fit of hybrids to any environment, and thus low fitness (Edmands 2007; Pekkala et al. 2012). Third, if populations have not diverged enough, there may be no benefit of outcrossing, as they are likely to harbor the same deleterious and beneficial alleles, and thus outcrossing will neither mask alleles that contribute to genetic load nor increase genetic variation.

This last situation is a challenge for management of small populations, for which the ideal populations for intraspecific hybridization are often not available. For example, reintroduced populations, such as the California condor ( *Gymnogyps californianus* ), often only have the captive bred population they were introduced from, or other populations reintroduced from that same captive bred population, for potential outcrossing (Ralls et al. 2000; Snyder and Snyder 2000; Walters et al. 2010). These might be so recently diverged that crossing among them may have little effect. As outcrossing can be challenging to execute at scale, experimentally confirming that it can have beneficial effects should be done prior to undertaking larger‐scale implementation.

The field of biological control faces similar predicaments to conservation of small populations and offers a unique and underutilized opportunity to study the effects of outcrossing of recently diverged populations. In biological control of weeds, specialized natural enemies, often insects, are introduced from a pest's native range to suppress invasive species (Cock et al. 2016; Heimpel and Cock 2018; Schwarzländer et al. 2018). Such biological control agents are particularly well‐suited for evaluating the effects of outcrossing of recently diverged populations since their population histories are well‐documented, and they can be reared and manipulated in controlled settings. Before release, their host range is studied in quarantine to evaluate whether they are specific to the targeted weed. In that time, populations can undergo genetic changes due to genetic drift, inbreeding and long quarantine rearing under artificial conditions. This can lead to the fixation of recessive deleterious alleles (Fauvergue et al. 2012; Bertin et al. 2017; Szűcs, Vercken, et al. 2019) as well as adaptation to laboratory conditions (Bertin et al. 2017; Szűcs, Vercken, et al. 2019). If approved for eventual release, new selection pressures and further demographic bottlenecks can compound these effects (Fauvergue et al. 2012; Hufbauer et al. 2013; Szűcs et al. 2017; Szűcs, Vercken, et al. 2019). Intentional hybridization of recently separated populations may help overcome these constraints and improve efficacy. Such biological control agents also offer model systems to manipulate the genetics of populations and test how hybridization influences heterozygosity and performance. Conducting the crosses provides direct information on the phenotypic effects of outcrossing, and population genomic analysis can provide insights into whether populations have diverged at all and are thus worth attempting to outcross, and whether crossing increases heterozygosity.

Here, we investigate the effects of intraspecific hybridization in *Hypena opulenta* Christoph (Lepidoptera: Erebidae), a specialist herbivore approved as a biological control agent to manage swallow‐wort vines (*Vincetoxicum* spp.) in North America. Swallow‐worts are invasive vines from Europe that negatively affect native plant and arthropod communities (Alred et al. 2022; Casagrande and Dacey 2007; Ditommaso et al. 2005; Ernst and Cappuccino 2005). We address three main questions. (1) Has a field population differentiated at the genomic level from the laboratory populations from which it arose? (2) How much diversity is harbored in the laboratory and field populations, and does hybridizing them increase heterozygosity? (3) How does hybridization affect performance in the laboratory and field relative to parental populations? We hypothesized that the laboratory and field populations would exhibit early signs of genetic divergence and that hybridization between them would lead to increased heterozygosity and improved performance. The recent shared origin of the populations allowed us to test whether populations separated for a relatively short period could diverge sufficiently for outcrossing to produce measurable effects, while reducing concerns associated with outbreeding depression.

## 2 Methods

### 2.1 Study System

The biological control agent *Hypena opulenta* was approved for release in Canada in 2013 and the United States in 2017 (Bourchier et al. 2019; Stewart 2017; Weed and Casagrande 2010). Larvae feed exclusively on swallow‐wort foliage across five instars before pupating in the soil and eventually emerging as adults. In *H. opulenta*, diapause, a state of halted development that allows insects to survive extreme conditions, occurs in the pupal stage and is dependent on photoperiod (Tauber et al. 1986; Weed and Casagrande 2010). In Michigan, *H. opulenta* can potentially complete two generations per year in the wild when released in spring, maximizing larvae exposure to longer daylight hours (Alred et al. 2022). While some individuals may produce a second generation, others enter diapause by mid‐summer, emerging the following year (Alred et al. 2022; Jones et al. 2020). After its 2013 field release in Canada, *H. opulenta*'s establishment was confirmed by 2015 in Ontario (Bourchier et al. 2019). In Michigan, however, overwintering success in the field wasn't verified until 2023 and its long‐term persistence in the region remains uncertain.

### 2.2 Moth Populations and Rearing

#### 2.2.1 Population Background

*Hypena opulenta* used for biological control in Northern America was originally collected in Donetsk, Ukraine in 2006. A colony was established at CABI EU‐CH (Delémont, Switzerland) using four pupae and 32 larvae (Weed et al. 2011). In 2008, *H. opulenta* were shipped to the University of Rhode Island (URI) where they were reared under quarantine conditions (Hazlehurst et al. 2012). In 2018, URI provided *H. opulenta* from their laboratory colony to Michigan State University (MSU; East Lansing, MI), where a laboratory population was founded with 18 females and 22 males. Hereafter this population is referred to as the laboratory population, as it had spent approximately 28 generations in culture by the beginning of our study.

In 2012, URI supplied *H. opulenta* to Canadian researchers, who initiated field releases in 2013. In 2020, individuals were obtained directly from this established Canadian field population (Bourchier et al. 2019) and used to found a population in the MSU laboratory with 15 females and 13 males. Though we reared this population in the laboratory, given its origin from an established field population, we refer to this as the field population. At the beginning of this study, this field population had been separated from the lab population for up to 14 generations in the field. Under laboratory conditions, more than two generations can be produced annually, whereas field populations are constrained by seasonal conditions inducing diapause and produce a maximum of two generations per year.

To generate an outbred population, reciprocal crosses between the laboratory and field populations were conducted in summer 2020. Pupae from the laboratory and field populations were sexed by measuring the distance between the genital slit and the anal slit and placed in separate cages by sex and population until adults emerged. Crosses were then created by placing these unmated adults no more than 2 days old in three separate mating cages: one with five lab females and three field males; a second with three lab females and two field males; and a third cage representing the reciprocal cross, with three field females and five lab males. Offspring from all crosses were combined to form the outbred population. This crossing design ensured reciprocal mating and thus genetic mixing from both directions. During and following crossing, the outbred population was maintained as a separate colony under the same rearing conditions as the laboratory and field populations, which is outlined below. Hereafter, this population is referred to as the outbred population.

#### 2.2.2 Plant Material

Both species of swallow‐wort (*V. rossicum* and *V. nigrum*) were historically used for colony rearing as *H. opulenta* does not discriminate between species for oviposition or feeding, and adult longevity and fecundity are similar on both hosts (Hazlehurst et al. 2012). However, by the start of the experiment in 2021, rearing and experimentation was exclusively conducted on *V. rossicum* due to its availability. Field collected plants of both *Vincetoxicum* spp. from infestations across Michigan including Grand Rapids and several towns in Oakland County, from sites where *H. opulenta* was never released, were transplanted into 1.14 L square plastic pots using SUREMIX all‐purpose perlite mix (Michigan Grower Products Inc., United States). Potted swallow‐wort was grown in the greenhouse at 26.6°C ± 4.9°C from March through September. In the fall, the potted plants were placed outside at the MSU Entomology Farm until the following spring to overwinter, which allowed them exposure to their natural seasonal cycle in Michigan and improved plant quality which decreased when plants were stored in the greenhouse year‐round. In March/April, the overwintering plants were brought back in the greenhouse and repotted, using the same SUREMIX potting soil, prior to use in rearing in subsequent seasons to improve plant health and avoid the introduction of pests. These plants were used for rearing the three populations (laboratory, field, outbred).

#### 2.2.3 Rearing and Population Maintenance

Rearing was conducted in the laboratory in mesh cages (40 × 40 × 60 cm, Restcloud) containing six potted swallow‐wort plants on a tray with a 0.6–1.3 cm layer potting soil. Rearing cages were kept in a laboratory at 22°C–25°C with ambient humidity under a 16:8 h L:D photoperiod provided by supplemental lighting (4 ft., 80 W; Barrina; Zhongshan, China).

The annual cycle during our research began in March with emergence of adults from diapause. Up to five male–female pairs of unmated adults were placed in each mesh cage and provided with honey water (1:5 ratio) in 59 mL cups with a cotton wick during mating and oviposition. We capped the density at five pairs of adults to avoid extremely high larval densities. Emerging larvae fed on the potted swallow‐wort and additional foliage was provided using cut stems of swallow‐wort in water picks (9 × 2.5 cm) as needed. Larvae fed in the mesh cages until 3rd–5th instar and were then moved to 2.4 L round plastic containers (HDX, Home Depot; United States) with mesh in the lids for ventilation. Larvae in these containers were fed swallow‐wort stems in water picks and provided paper towels for pupation. Containers were maintained in a laboratory at 22°C–25°C with ambient humidity under 16:8 h L:D grow lights and checked daily to every other day for pupae. Under high volume conditions, some larvae were allowed to pupate in mesh cages.

Pupae were collected from plastic containers and mesh cages, sexed, and placed in same‐sex groups of eight or less into 0.35 L transparent round plastic containers with vermiculite (Vigoro; United States) moistened with distilled water. During the continuous rearing season, these containers of pupae were placed on the benchtop under 16:8 h L:D lights until emergence. In August, pupal containers were placed in incubators under 12:12 h L:D at 20°C to induce diapause. The temperature was lowered by 5°C each month until 5°C was reached and maintained. The containers were remoistened once a month until placement under 16:8 h L:D and 22°C–25°C in early spring. The laboratory population was maintained using the above protocol from 2018 to the start of experiments in 2021 and 2022, and the same protocol was used for the field and outbred populations following their establishment in the lab in 2020.

### 2.3 Molecular Structure and Genetic Diversity

#### 2.3.1 DNA Extraction, Sequencing, and Bioinformatics

*Hypena opulenta* adults from the laboratory (*n* = 15 total from 2021), field (*n* = 15 total, *n* = 8 from 2020 and *n* = 7 from 2021) and the outbred population (*n* = 20 total, *n* = 10 from 2020 and *n* = 10 from 2021) were saved and stored dry at −80°C. Because the individuals were taken as adults for storage, and sex is difficult to determine at that stage, sex was not recorded (and as noted below, sex chromosomes were thus excluded from analysis). The Novogene Corporation Inc. (Durham, NC, USA) performed DNA extraction, library preparation, whole genome sequencing and sequence alignment. Whole genome sequencing libraries were prepared using the Illumina Standard 350 bp DNA library. DNA from each individual was extracted and prepared as a separate library and individuals from the three populations were not pooled prior to library construction. Libraries were checked with Qubit and real‐time PCR for size detection and distribution. Following individual library preparation and indexing, libraries were pooled for multiplexed sequencing by Novogene on NovaSeq 6000 (Paired‐End 150 bp).

Novogene completed a standard bioinformatic analysis including data quality control, alignment with a reference genome and statistics of sequencing depth and coverage. The sequencing error rate for all individuals was 0.03% with the percentage of Phred‐scaled quality scores greater than 30 all above 91%. Reads were aligned to the *Hypena proboscidalis* reference genome available through NCBI (genome assembly: ilHypProb1.1; assembly accession: GCA_905147285.1; Boyes and Holland et al. 2021). The mapping rate ranged from 28.55% to 30.43% and average depth ranged from 16.10× to 21.46×.

#### 2.3.2 Population Structure

To evaluate differentiation among the sampled populations, PCAngsd (v.1.21) was used to calculate individual allele frequencies and their covariance for structured populations using principal component analysis (PCA) using genotype likelihoods (Meisner and Albrechtsen 2018). PCAngsd requires genotype likelihoods files to be input in a beagle format; therefore, BAM files were converted to beagle files using ANGSD (v.0.935) (Korneliussen et al. 2014), which is designed to estimate genotype likelihoods directly from sequencing data while accounting for genotype uncertainty. Conversion was performed using the parameters ‐doGlf 2, ‐GL 1, and ‐SNP_pval 1e‐6 to work with sites with this *p*‐value < 1e‐6. During conversion, the regions were again filtered to include autosomes 1–30 for consistency. Individual allele frequencies were calculated by simply running PCAngsd by inputting the beagle file.

#### 2.3.3 Genome‐Wide Diversity

ANGSD was used to calculate the global, genome‐wide nuclear heterozygosity of individual samples. This software uses the entire genome and incorporates genotype uncertainty by using genotype likelihoods (GLs). This method is particularly useful for non‐model organisms with low to medium coverage (Korneliussen et al. 2014).

For each sample a reference genome is provided as the ancestral state. We first took the site allele frequency likelihood, which is based on individual genotype likelihoods assuming Hardy–Weinberg equilibrium, by using the ‐doSaf 1 option. Filters were used since the input files are raw alignments (BAM and FASTA files) and were applied concurrently within a single ANGSD command during genotype likelihood estimation. The filter ‐C 50 was used to adjust mapping quality for excessive mismatches. Then reads were removed if mapping quality fell below 30 (‐minMapQ 30), minimum base quality score was below 20 (‐minQ 20), they were flagged if above 255 (‐remove_bads), if location was not unique during mapping (‐uniqueOnly), and filter for pairs of reads with both mates mapped correctly (‐only_proper_pairs). The regions used were filtered (‐rf) to include all 30 autosomes and exclude the sex chromosome. Genotype likelihoods were calculated using ‐GL 1 (SAMtools method). The result was then used to obtain the maximum likelihood estimate of the SFS (Site Frequency Spectrum) by using the subprogram realSFS. Heterozygosity was then calculated by using the data in the SFS by taking the number of heterozygous sites and dividing it by the total number. This method was adapted from de Jager et al. (2021).

Heterozygosity values were assessed for normality using a Shapiro–Wilk test then modeled with a one‐way ANOVA using the *stats* package (R Core Team 2023). Population (lab, field, outbred) was used as the fixed effect. A Q–Q plot and Levene's test were used to assess model assumptions from the *car* package (Fox and Weisberg 2019).

### 2.4 Laboratory Performance

To compare the performance of the three *H. opulenta* populations (laboratory, field and outbred), we quantified how many pupae were produced by individual females as a proxy for fitness. Pupal production integrates multiple fitness components, including reproductive output and survival through the larval stage. Additionally, because larvae consume swallow‐wort foliage prior to pupation, survival to the pupal stage is closely associated with potential biocontrol impact. Individual unmated females, no more than 3 days old, were allowed to mate and lay eggs for 72 h in rearing cages. One to two males were used for mating based on availability, but the numbers were kept consistent in each temporal block. We exclusively used *V. rossicum* for egg laying which was transplanted from field sites in Oakland County, MI to pots in the greenhouse in the spring of 2021 and 2022. For logistical reasons these experiments were conducted at two locations (laboratory and greenhouse) in staggered time frames associated with the consecutive generational cycles of *H. opulenta* (blocks) over a two‐year period (2021 and 2022). Overall, the number of replications (individual females tested) for each of the three treatments (laboratory, field and outbred populations) ranged between 68 and 87. Two blocks were set up both in 2021 and in 2022. In 2021, both blocks were tested in the laboratory. In 2022, the first block was set up in the greenhouse and the second and third blocks in the laboratory. In the greenhouse experiment the temperature was 25.6°C ± 4.8°C and beyond natural daylight hours artificial lights were used to ensure 16:8 L:D photoperiod. In the laboratory the cages were kept under ambient temperature and humidity with artificial lighting set to 16:8 h L:D. The number of replications for each year, temporal block and location are summarized in Table 1.

**TABLE 1:** Summary of the years, blocks and replicates for the laboratory experiment.

Year	Block	Population	Generation	Number of replicates	Location
2021	1	Field	F3	4	Lab
		Lab	F10	4	Lab
		Outbred	F3	4	Lab
	2	Field	F4	4	Lab
		Lab	F11	4	Lab
		Outbred	F4	4	Lab
2022	1	Field	F6	39	Greenhouse
		Lab	F13	33	Greenhouse
		Outbred	F6	37	Greenhouse
	2	Field	F7	15	Lab
		Lab	F14	20	Lab
		Outbred	F7	23	Lab
	3	Field	F8	6	Lab
		Lab	F15	13	Lab
		Outbred	F8	19	Lab

### 2.5 Field Performance

To compare the performance of the three populations of *H. opulenta* under field conditions, adults were released in cages set up in a natural infestation of *V. rossicum* at a field site in Oakland County, MI (42.7087042, −83.5732006). Cages were square frames made of polyvinyl chloride and covered with a fine white mesh that were staked to the ground over naturally growing swallow‐wort (60 × 60 × 60 cm; Gardeners Advantage). Cages were set up within a ~500‐square meter infestation in early June of 2023. In each cage, three types of annual flower plugs were planted (one each of marigold, begonia, and impatiens) to provide nectar for adult moths. There were 14 replicates for each population treatment (field, lab, and outbred), totaling 42 cages. A total of five 1–5 days old female and five male adult moths were released in each cage on June 14th, 2023. Swallow‐wort stem counts within each cage were taken before release as a measure of host plant availability. Percent defoliation was quantified from leaf tissue consumption on five randomly chosen stems in each cage once on July 10th, 2023, when fifth instar larvae were present, and pupation had started. Percent defoliation is used as a proxy for fitness for each population treatment. The number of 2nd generation adults emerged in each cage was checked weekly after the start of pupation until the end of August. Adults were released from the cage upon each monitoring date to avoid double counts later.

### 2.6 Statistical Methods of Performance Experiments

All analyses were performed in R 4.3.0 (R Core Team 2023). We used the *DHARMa* package to calculate the residuals to assess best model fit for all non‐parametric models. This package assesses the model in several ways including Q–Q plot, the Kolmogorov–Smirnov test (K.S. test), dispersion testing, and zero‐inflation testing. Chi‐squared values were provided for mixed models using the Anova function from the *car* package (Zeileis and Hothorn 2002). All post hoc pairwise comparisons were performed using the *emmeans* package and were adjusted using Tukey's method (Lenth et al. 2021).

#### 2.6.1 Laboratory Performance

In this experiment, we assessed if a female succeeded or failed in producing any pupae and then the number of pupae produced by each female. Statistically, we compared the production of pupae by population in two ways. First, we evaluated whether any pupae were produced (hereafter “production success”) with a generalized linear mixed model with a binomial distribution using the *lme4* package (Bates et al. 2015). If no pupae were produced, then the replicate was assigned a “0”. If one or more pupae were produced, then the replicate was assigned a “1”. The model included population (lab, field, outbred), year (2021, 2022), and location (laboratory, greenhouse) as fixed effects. We included an interaction between population and year to assess whether population performance changed over time. We used replicate nested within block as a random effect to account for variation in replicate setup timing within blocks.

Using the same fixed and random effects as above, we evaluated the number of pupae produced per female using a generalized linear mixed model via the *glmmTMB* package with a Poisson distribution (Brooks et al. 2017). The *glmmTMB* package allows for a zero‐inflation parameter to be applied to a model with a Poisson distribution. The zero‐inflation parameter was set to vary by location such that the probability that observations had structural zeros depended on population.

#### 2.6.2 Field Performance

In this experiment, we measured if a group of five females succeeded or failed in producing any larvae by noting evidence of larval feeding. Defoliation was quantified by visually estimating the percentage of tissue consumed for each leaf on a stem. Estimates were summed across all leaves and divided by the maximum possible defoliation for that stem (100% × total number of leaves) to calculate percent defoliation. We also measured the number of second‐generation adults produced by each population. First, we used the presence or absence of larval feeding to compare performance between the three populations. If a cage exhibited no feeding, then the replicate was assigned a “0”. If a cage exhibited any feeding, then the replicate was assigned a “1”. A generalized linear mixed model was used with a binomial distribution with the *glmmTMB* package in R (Brooks et al. 2017). We used population (field, lab, and outbred) as the fixed effect and swallow‐wort stem density as the random effect.

In addition, we compared total defoliation per stem as a proportion (between 0 and 1) using a generalized linear mixed model with a beta distribution in the *glmmTMB* package in R for all cages (Brooks et al. 2017). This package allows a zero‐inflation parameter that permits zeros in the data because beta distributions are bound between greater than zero and less than one yet is often zero or one inflated. This approach allowed us to set the zero‐inflation and dispersion parameter to vary by population. We used population (field, lab, and outbred) and swallow‐wort stem density as a fixed effect and, as the random effect, we assigned a unique cage identification to each cage to account for non‐independence of stems within a cage. One replicate in the field population was missed during data collection, and therefore *n* = 13 for this treatment while *n* = 14 for the other populations.

Adult emergence success by treatment was evaluated by assigning cages with no emergence a “0” and one or more adult emergence a “1”. A generalized linear model was used, from the *lme4* package, with a binomial distribution with population and emergence date as fixed effects (Bates et al. 2015). The number of adults that emerged by each treatment was compared with a generalized linear model with a Poisson distribution, from the *lme4* package (Bates et al. 2015). We used two fixed effects, population and emergence date. Emergence was only recorded on two dates with only one cage with one adult emergence at the later date. In a separate analysis, only population was used as a fixed effect, and the number of emerging adults was summed for the two dates. Two assessments were conducted because emergence was not fully synchronized and emergence timing may influence opportunities for reproduction prior to seasonal diapause.

## 3 Results

### 3.1 Population Structure and Molecular Genetic Diversity

#### 3.1.1 Population Structure

**FIGURE 1:**
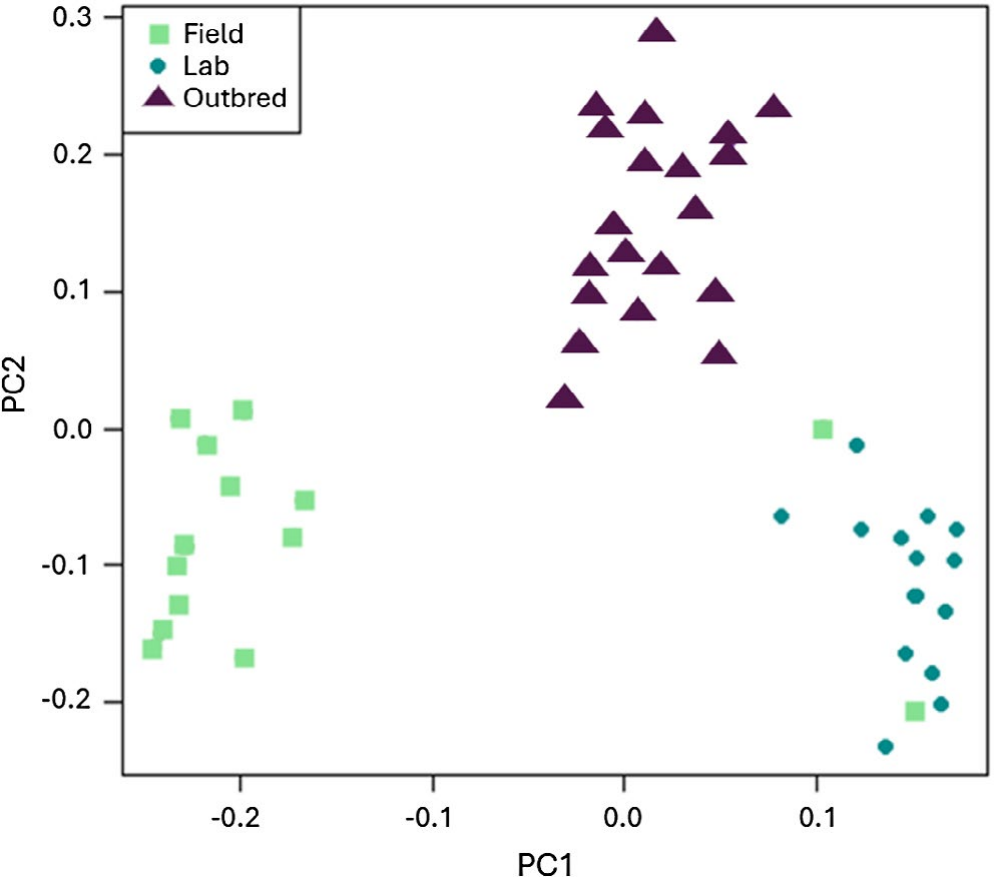
Population structure using individual allele frequencies of *Hypena opulenta* from the field, the lab, and outbred populations. The principal component analysis (PCA) plot is the percentage of variation explained by each principal component.

The population structure of *H. opulenta* based on the principal component analysis of allele frequencies displays three distinct populations that correspond to the three population treatments (Figure 1). Two individuals from the field population deviate from the group by clustering more closely with the lab population. The outbred and field populations occupy opposite ends of the first principal component. The lab population clusters closer to the outbred population yet is still distinct as its first principal component is positive. Populations seem to be primarily divided by the first principal component; however, some individuals in each population are more similar by the second principal component, falling closer to zero.

#### 3.1.2 Genome‐Wide Diversity

**FIGURE 2:**
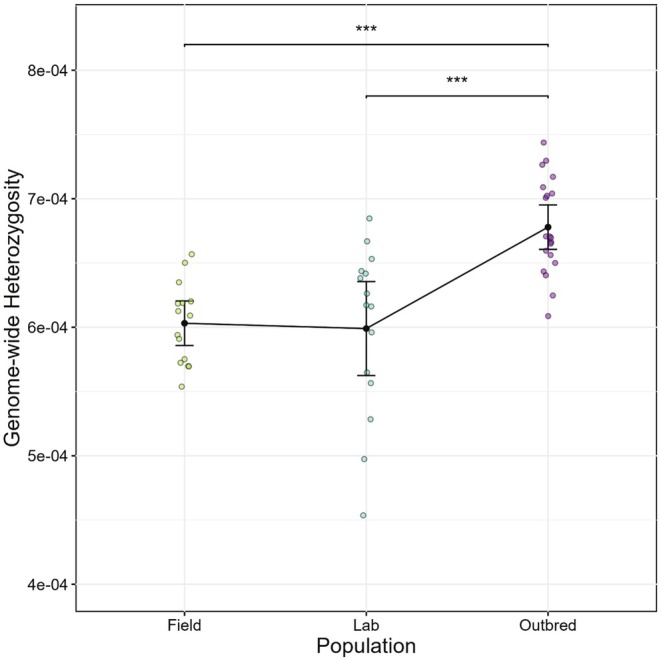
Mean and 95% confidence interval (vertical brackets) of genome‐wide heterozygosity by population (field, lab, outbred). Observations have been jittered to show individual points. Asterisks indicate significant differences between treatments at the 0.05 (*), 0.01 (**), 0.001 (***) levels.

The mean global heterozygosity of the outbred population was significantly higher than the field and the lab populations' (*F* = 16.59, df = 2, *p* < 0.0001) (Figure 2). Mean heterozygosity was 12% higher in the outbred population than in the field population (pairwise comparison: *p* = 0.0001) and 13% higher than the lab population (pairwise comparison: *p* < 0.0001). Heterozygosity of the field and the lab population did not differ (*p* = 0.968).

### 3.2 Laboratory Performance

The probability that at least one pupa would be produced was not affected by population (*χ* ^2^ = 2.12, df = 2, *p* = 0.35) or year (*χ* ^2^ = 0.17, df = 1, *p* = 0.68), nor the interaction (*χ* ^2^ = 3.81, df = 2, *p* = 0.15). However, it was affected by location (*χ* ^2^ = 10.67, df = 1, *p* = 0.001). The odds of successfully producing at least one pupa were 3.5 times higher in laboratory conditions than in greenhouse conditions (pairwise comparison: *p* = 0.001).

**FIGURE 3:**
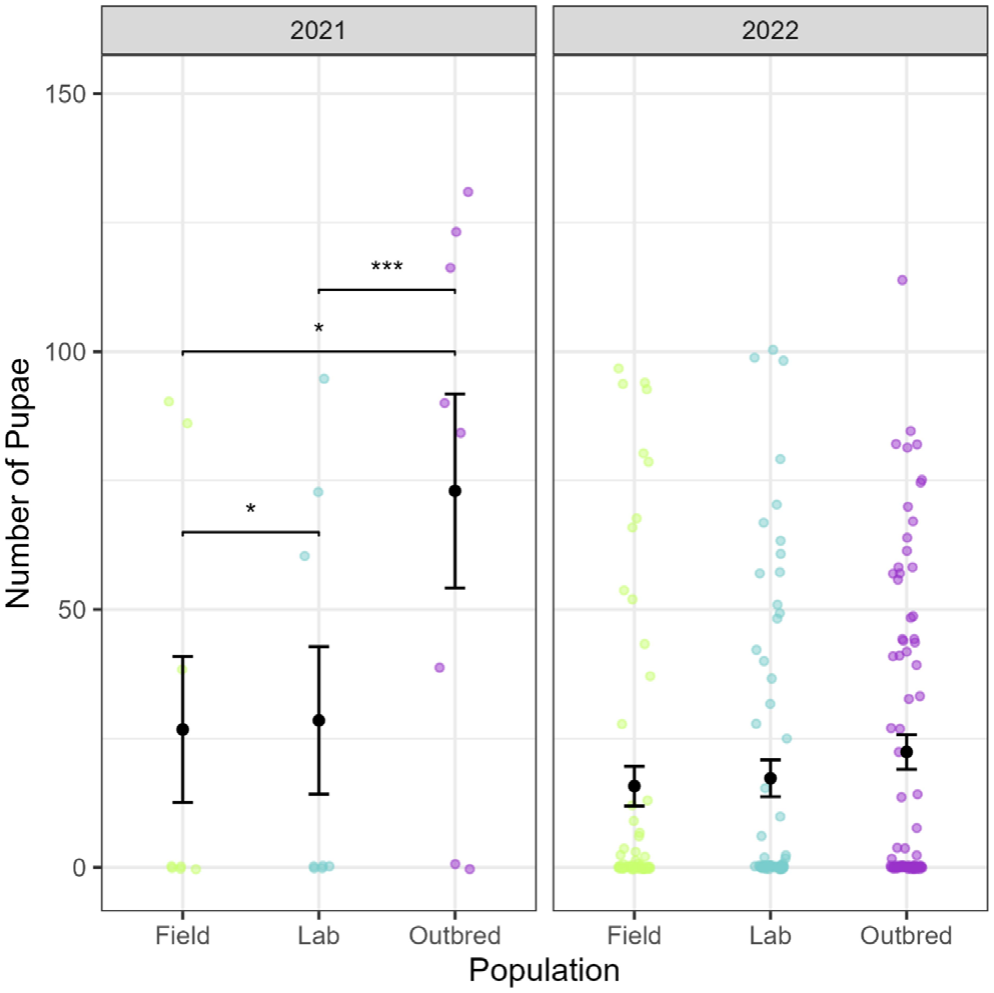
Means and standard error (vertical brackets) of the number of pupae produced per female by population (field, lab, outbred) and separated by year (2021, 2022). Observations have been jittered to show individual points. Asterisks indicate significant differences between treatments at the 0.05 (*), 0.01 (**), and 0.001 (***) levels.

The number of pupae produced was affected by population (*χ* ^2^ = 31.61, df = 2, *p* < 0.0001) and the interaction between population and year (*χ* ^2^ = 17.27, df = 2, *p* < 0.001), but not by year alone (*χ* ^2^ = 0.0058, df = 1, *p* = 0.94) (Figure 3). In 2021, the outbred population produced 1.6 times more pupae than the lab population (pairwise comparison: *p* < 0.0001) and 1.25 times more pupae than the field population (pairwise comparison: *p* < 0.05). Differences between the parental populations were comparatively small (pairwise comparison: *p* = 0.05). No pairwise population comparisons were significant in 2022. Laboratory conditions produced 3.7 times more pupae than greenhouse conditions (pairwise comparison: *p* < 0.0001).

### 3.3 Field Performance

The probability that a cage would produce at least one larva (i.e., visible feeding damage) was not significantly different between the populations (*χ* ^2^ = 3.84, df = 2, *p* = 0.15) (pairwise comparisons: all *p* > 0.05) but was marginally significant by swallow‐wort stem density (*χ* ^2^ = 3.90, df = 1, *p* = 0.048). However, the outbred population had the highest probability of success (99.5%), the lab population the second‐highest success probability (97.9%) and the field population the lowest success probability (94.6%) in terms of replicated field cages showing any feeding damage. Post hoc pairwise comparisons were not significant.

**FIGURE 4:**
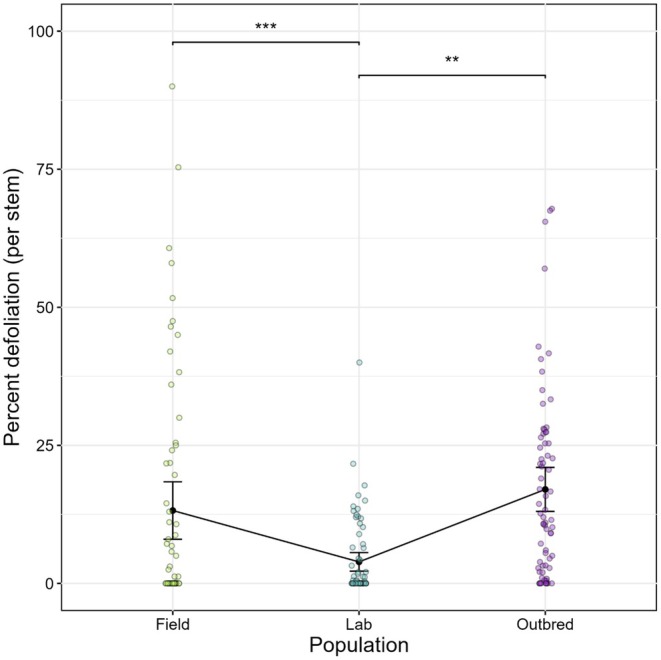
Mean and 95% confidence intervals (vertical brackets) of the percent of leaf matter defoliated per stem by population (field, lab, outbred). Observations have been jittered to show individual points. Asterisks indicate significant differences between treatments at the 0.05 (*), 0.01 (**), 0.001 (***) levels.

Percent defoliation was affected by population (*χ* ^2^ = 12.55, df = 2, *p* < 0.001) (Figure 4) but not by swallow‐wort stem density (*χ* ^2^ = 0.62, df = 1, *p* = 0.43). The outbred population had the highest mean defoliation (0.17 [0.13, 0.21], mean [95% confidence interval], *n* = 14) followed by a lower mean defoliation of the field population (0.13 [0.08, 0.18], *n* = 13), but these differences were not significant. The outbred population produced significantly more defoliation than the lab population (0.04 [0.02, 0.06], *n* = 14) (pairwise comparison: *p* < 0.01). The field population also had higher rates of defoliation when compared to the lab population (pairwise comparison: *p* < 0.001).

The probability that at least one adult would emerge was not affected by population (*χ* ^2^ = 4.56, df = 2, *p* > 0.05). The probability of emergence depended on date (*χ* ^2^ = 15.10, df = 1, *p* < 0.001). The probability that a second generation of adults would emerge was 1% for the field and the lab populations, and 5% for the outbred population (pairwise comparisons were not significant). Adults were 22% more likely to emerge by August 8 than August 16 (pairwise comparison: *p* < 0.01).

Significantly more second generation adults emerged for the outbred population (4.02 [1.30, 17.55], *n* = 14, *N* = 13; mean [95% confidence interval], *n* = number of replicates, *N* = number of emerged adults) compared to the field population (0.012 [0.0006, 0.069], *n* = 13, *N* = 3) and lab (1.24 [0.27, 6.29], *n* = 14, *N* = 4) (population: *χ* ^2^ = 7.97, df = 2, *p* < 0.05). The number of adults that emerged depended on date (*χ* ^2^ = 19.79, df = 1, *p* < 0.001). The outbred population produced 3.3 times more adults than the field population (pairwise comparison: *p* = 0.076) and 2.3 times more adults than the lab population (pairwise comparison: *p* = 0.098); thus, neither pairwise comparison was significant. Ninety‐seven percent of the adults emerged by August 8th, 2023, compared to August 16 (pairwise comparison: *p* < 0.01). When dates were combined to assess the number of emerged adults, the results for population differences were similar, and the pairwise comparison between the outbred population and the lab population became marginally significant (pairwise comparison: *p* = 0.057).

## 4 Discussion

This study demonstrates that intentional intraspecific hybridization can significantly enhance genetic diversity and improve several performance metrics in a classical biocontrol agent, even when parental populations have diverged recently. Despite a short separation and similar levels of heterozygosity, the laboratory and field populations developed distinct genetic structures. Hybridization between them led to increased heterozygosity and improved performance across multiple years and experimental settings, although the magnitude of these effects varied among performance measures. These results highlight the potential of using neutral genetic diversity as a predictor of performance and suggest that intentional hybridization may provide a short to medium term boost of hybrid vigor that improves the efficacy of biological control agents.

Our results show that distinct population structure developed between the laboratory and field populations of *H. opulenta*, despite only 7 years (or 14 generations) of separation. This demonstrates that meaningful genetic divergence can arise over short timescales, even among recently isolated populations. Such divergence is not limited to field conditions since laboratory populations can also become genetically different. For example, *Aedes aegypti* laboratory strains exhibit population structure that is more strongly associated with their specific laboratory environments than with their shared wild origin, indicating that even minor environmental differences can drive divergence (Gloria‐Soria et al. 2019). Similar patterns have been observed in *Drosophila subobscura* where laboratory populations derived from a common wild source developed significant genetic differences within 14 generations due to genetic drift and adaptation to laboratory conditions before divergence plateaued (Simões et al. 2008). In *Gambierdiscus caribaeus*, a marine microalga, notable shifts in genetic structure occurred within just 2 months in response to environmental changes such as salinity, underscoring how quickly allele frequencies can be shaped by selection and drift (Sassenhagen et al. 2018). Collectively, these examples illustrate that genetic drift, inbreeding, and adaptation to distinct environments can rapidly generate population structure—processes that are highly relevant to biological control agents reared in captivity and released into novel environments.

We found that hybridizing two inbred populations of *H. opulenta*—each with similarly low levels of genome‐wide heterozygosity—resulted in a hybrid population with significantly higher heterozygosity. This pattern, in which hybrids exceed their parental populations in genetic diversity, likely reflects the independent evolutionary trajectories of the parental lineages. A similar phenomenon was observed in *Oncorhynchus clarkii lewisi* (westslope cutthroat trout), where hybridization among geographically and demographically distinct, low‐diversity populations led to offspring with elevated heterozygosity (Feuerstein et al. 2024). This was attributed to each source population having fixed different alleles through drift, inbreeding, or local adaptation, and the resulting hybrids capturing complementary variation from both lineages (Feuerstein et al. 2024). In *Anolis sagrei* (Cuban brown anole) invasive populations arising from admixture among genetically distinct native sources produced hybrids with greater genetic diversity than any single lineage, due to recombination of previously isolated genotypes once geographic barriers were removed (Bock et al. 2021). Similarly, in the egg parasitoid *Trichogramma dendrolimi* (a biocontrol agent for lepidopteran pests), outcrossing among genetically distinct laboratory lines increased fecundity and body size, further supporting the idea that admixture between differentiated lineages can improve performance through increased genetic variation (Liang et al. 2025). Although the genetic mechanisms differ between the haplodiploid *T. dendrolimi* and the diploid *H. opulenta*, both studies suggest that introducing genetic variation through outcrossing can improve fitness‐related traits. In our study, the laboratory and field populations of *H. opulenta* experienced different environmental conditions, likely resulting in divergence at different genomic regions. Hybridization between them effectively combined these differentiated genomes, producing increased genome‐wide heterozygosity. This finding reinforces evidence from other systems that hybridization can restore or increase genetic diversity even when both parental populations are inbred, provided they have been shaped by different evolutionary forces.

In our laboratory experiments, outbred females produced more pupae after the initial outcrossing, indicating enhanced fecundity and/or improved larval survival to the pupal stage. This performance boost could have been due to hybridization increasing heterozygosity and masking deleterious alleles present in the inbred parental populations. Despite having successfully established in the field, the field population had poor performance under laboratory conditions. This can be attributed to a combination of pre‐release factors, including adaptation to culture and heterozygosity loss during mass‐rearing as population sizes fluctuated. Moreover, this population faced novel biotic and abiotic conditions upon field release and thus, novel selection pressures. Adaptation to the field conditions could have led to further reductions in heterozygosity and low population sizes if there was a hard selection event that resulted in high mortality of suboptimal or unsuitable genotypes in the novel environment. Conversely, the lab population's low performance likely stems from extended rearing over 45 generations, during which genetic drift and inbreeding occurred, despite likely adaptation to lab conditions.

The hybrid individuals tested in the laboratory were F3 to F8, whereas those in the field experiment were F9 hybrids, a generation in which heterosis is often expected to decline. Despite this, the outbred population exhibited improved performance in both laboratory and field experiments, suggesting that the benefits of intraspecific hybridization can persist across multiple generations in this system, as seen in other taxa (Szűcs et al. 2014; Wagner et al. 2017). However, the magnitude of these benefits varied among years and performance measures, with signs of waning performance in metrics such as pupal production. The strongest laboratory effects occurred in the earlier years of the experiment, whereas field experiments with later‐generation hybrids continued to show improved performance. Continued rearing under laboratory conditions may eventually erode the benefits further, raising important questions about the optimal timing for field release of laboratory‐created hybrid populations.

In field conditions, the field population outperformed the lab population, consistent with expectations and highlighting the potential pitfalls of evaluating biocontrol agents solely under lab conditions, which often involve mass‐rearing regimes. Overall, the outbred population had the best performance in the field, particularly in terms of larval feeding and second‐generation adult emergence. Interestingly, defoliation rates were similar between the outbred and field populations, suggesting that adaptation to field conditions can yield comparable benefits to hybridization in some traits. Together, these findings indicate that hybridization can enhance performance in biocontrol agents, and that genome‐wide heterozygosity may serve as a useful predictor of field success in the absence of known functional markers.

Although hybridization is well documented in naturally admixed populations, relatively few studies have assessed field performance of hybrids alongside genetic data (Abbott et al. 2016). Most existing work focuses on naturally occurring hybridization, as in the weed biological control agent *Longitarsus jacobaeae*, where hybrid populations outperformed parental lines in controlling tansy ragwort (Szűcs, Salerno, et al. 2019). Similar patterns are observed in invasive species; for example, in *Anolis sagrei* , hybridization was associated with increased body size and colonization success (Kolbe et al. 2007). Genetic admixture has also been linked to enhanced performance in invasive plants like *Spartina alterniflora* , *Mimulus guttatus* , and *Centaurea solstitialis* (Barker et al. 2017; Li et al. 2018; Qiao et al. 2019). To our knowledge, no published studies have moved beyond correlative evidence to experimentally perform intraspecific hybridization and test hybrid performance in the field. Our study fills this gap by using controlled hybrid crosses and standardized introductions, eliminating confounding factors such as differences in propagule pressure or introduction history (Baker et al. 2003; Dlugosch et al. 2015; Szűcs et al. 2017).

In conservation and restoration, the importance of genetic diversity is well‐established (Chapman et al. 2009; DeWoody et al. 2021; Hufford and Mazer 2003), yet it remains underutilized in biological control. Poor establishment and limited impact in many biocontrol programs may stem from genetic constraints during pre‐release rearing. As access to novel source populations becomes increasingly restricted by regulatory and logistic barriers, interest has grown in improving the performance of existing biological control populations through genetic management (Chattington et al. 2025). While unintentional intraspecific hybridization has occasionally improved performance, for example, in the predatory ladybird *Cryptolaemus montrouzieri* (Li et al. 2018), intentional crosses are rare, partly due to concerns about altered host‐specificity (Hoffmann et al. 2002; Mathenge et al. 2010; Szűcs, Vercken, et al. 2019). However, there is no evidence that intraspecific hybridization would result in either host shifts or host range expansion and fewer than 1% of weed biocontrol agents exhibit non‐target effects (Clark et al. 2023; Dieckhoff et al. 2017; Hinz et al. 2020; Szűcs, Vercken, et al. 2019).

Beyond biological control, these insights are relevant to conservation programs that use assisted gene flow or genetic rescue to increase genetic diversity and population viability. Our results demonstrate that populations separated for relatively short periods may diverge sufficiently for intraspecific hybridization to increase heterozygosity and performance, supporting the idea that beneficial effects of hybridization can arise even among populations with a recent shared origin. Strategic intraspecific hybridization that considers the potential risks of outbreeding depression may be a useful tool for increasing genetic diversity and improving population performance in some conservation contexts, particularly in the face of habitat loss, fragmentation, and environmental variability (Dawson et al. 2011; Urban 2015). Continued research into the relationship between genetic variation and performance, both in controlled and natural environments, remains vital for addressing applied challenges in invasion biology, conservation, and restoration.

## Funding

This work was supported by the Michigan Department of Natural Resources Michigan Invasive Species Grant Proposal (award # IS18‐2006 and IS‐21‐0284).

## Conflicts of Interest

The authors declare no conflicts of interest.

## Data Availability

Performance data will be made available in Ag Data Commons, and raw sequence reads for each individual will be made available through NCBI.
